# HSCCC Separation of the Two Iridoid Glycosides and Three Phenolic Compounds from *Veronica ciliata* and Their in Vitro Antioxidant and Anti-Hepatocarcinoma Activities

**DOI:** 10.3390/molecules21091234

**Published:** 2016-09-15

**Authors:** Qiuxia Lu, Yiran Sun, Yueyue Shu, Shancai Tan, Li Yin, Yiran Guo, Lin Tang

**Affiliations:** 1 Key Laboratory of Bio-Resources and Eco-Environment of Ministry of Education, College of Life Sciences, Sichuan University, Chengdu 610065, Sichuan, China; yinlin0827@163.com (Q.L.); 18202863885@163.com (Y.S.); shuyueyue888@163.com (Y.S.); m15828106432@163.com (S.T.); yinli0130@gmail.com (L.Y.); guoyr123456789@163.com (Y.G.); 2 National and Local Joint Engineering Laboratory for Energy Plant Bio-Oil Production and Application, Chengdu 610065, Sichuan, China

**Keywords:** separation, iridoid glycosides, phenolic compounds, *Veronica ciliata*, high-speed countercurrent chromatography, antioxidant, anti-hepatocarcinoma

## Abstract

Five main compounds, including two iridoid glycosides (catalposide, verproside) and three phenolic compounds (luteolin, 4-hydroxy benzoic acid, 3,4-dihydroxy benzoic acid), were separated and prepared from the crude extract of *Veronica ciliata* by high-speed countercurrent chromatography. *n*-Hexane/*n*-butanol/water (1.5:5:5, *v*/*v*/*v*) was used for the separation of catalposide and verproside. *n*-Hexane/*n*-butanol/water (3:2:5, *v*/*v*/*v*) was used for the separation of luteolin, 4-hydroxy benzoic acid and 3,4-dihydroxy benzoic acid. The head-to-tail elution mode was used with a flow rate of 5.0 mL/min and a rotary speed of 800 rpm. Finally, a total of 1.28 mg luteolin, 6 mg 4-hydroxy benzoic acid, 2 mg 3,4-dihydroxy benzoic acid, 2 mg verproside and 10 mg catalposide with purities of 98%, 99.1%, 99.5%, 99.8% and 99%, respectively, were obtained from 200 mg of crude extract. In addition, their structure was identified using MS, ^1^H-NMR and ^13^C-NMR. To the best of our knowledge, this is the first report of the separation and purification of iridoid glycosides and phenolic compounds from *V. ciliata* by high-speed countercurrent chromatography (HSCCC). Among these compounds, luteolin, 4-hydroxy benzoic acid and 3,4-dihydroxy benzoic acid were separated from *V. ciliata* Fisch. for the first time. The results of the antioxidant activity show that protocatechuic acid and luteolin have strong antioxidant activity compared to 2,6-di-*tert*-butyl-4-methylphenol (BHT) and vitamin C (Vc). Five compounds also exhibited strong anti-hepatocarcinoma activities.

## 1. Introduction

*Veronica ciliata* Fisch., belonging to the family Scrophulariaceae, is a traditional Tibetan medicine found in western China. This has been widely used for the treatment of hepatitis, cholecystitis, rheumatism and urticaria. About 100 types of Tibetan medicine preparations contain *V. ciliata* Fisch., particularly those used to cure hepatitis. Thus, *V. ciliata* Fisch. has an irreplaceable effect on the treatment of hepatitis. The crude extracts of *V. ciliata* Fisch. have been reported to have strong antioxidant activities and a significant protective effect against acute hepatotoxicity induced by CCl_4_ [1,2].

Previous phytochemical studies have shown that iridoid glycosides and phenolic compounds are the main compounds in *V. ciliata* Fisch. Pharmacological studies have revealed that the iridoid glycosides separated from *V. ciliata* Fisch. have strong inhibitory activity on HepG2 cell proliferation [2,3]. Moreover, phenolic compounds possess strong antioxidant, anti-hepatocarcinoma, antimicrobial and anti-inflammatory activities. For further studies, a large amount of iridoid glycosides and phenolic compounds is urgently needed as the chemical reference standards and for further anti-hepatocarcinoma studies. Therefore, it is necessary to establish a rapid and effective method to separate these active chemical components [4,5,6,7,8].

Conventional column chromatography and thin-layer chromatography are usually used to separate iridoid glycosides and phenolic compounds from *V. ciliata* Fisch. However, these methods are time-consuming and suffer from irreversible adsorption, waste of solvent, low recovery and high cost [9,10,11].

High-speed countercurrent chromatography (HSCCC) is a liquid-liquid partition chromatography based on the different partition coefficients of the compound in two immiscible phases to achieve separation. HSCCC has some advantages over traditional liquid-solid chromatography, such as the elimination of irreversible adsorption and denaturation of the sample on the solid stationary phase, as well as high recovery and efficiency. HSCCC is also efficient for the separation of the target compounds from a crude extract without pretreatment and high sample loading [12,13].

Successful HSCCC separation of natural products from other medicinal plants has been reported [14,15]. However, the application of HSCCC for the isolation of the main active compounds from *V. ciliata* Fisch. has not been reported. In this report, we successfully carried out the preparative separation and purification of two iridoid glucosides and three phenolic compounds from *V. ciliata* Fisch. for the first time by HSCCC. The HSCCC separation conditions, including a two-phase solvent system, flow rate and the revolution speed of the separation column, were optimized. The structure of the compounds was identified by MS, ^1^H-NMR and ^13^C-NMR analyses. The antioxidant activity of the five compounds was preliminarily evaluated by DPPH, ABTS radical-scavenging capacity and reducing power assays. The anti-hepatocarcinoma activities were also evaluated against HepG2 cell lines by the CCK-8 method.

## 2. Results

### 2.1. Optimization of UPLC Analysis

To the best of our knowledge, this is the first report on establishing a UPLC/PDA mode for the analysis of the extract of *Veronica ciliata* Fisch. During the optimization of the separation conditions, the system conditions, including the mobile phase composition (acetonitrile/water and methanol/water), with or without 0.1% formic acid, the gradient program (gradient time, gradient shape) and column conditions, were investigated [16]. Water + 0.1% formic acid (A) and can (B) were used in the optimized gradient program as follows: 10%–25% B (0–0.8 min), 25%–35% B (0.8–2.4 min), 35%–95% B (2.4–2.8 min), 95% B (2.8–4 min). Under this condition, all of the iridoid glycosides and phenolic compounds reached base-line separation within 4 min. 

### 2.2. Selection of HSCCC Experimental Conditions

The selection of a suitable two-phase solvent system is the most important step for separating compounds by HSCCC. Several factors should be considered: (1) the structure and polarity of the target compound: iridoid glycosides and phenolic acids are highly polar compounds and easily dissolve in water; and the crude sample used for the separation of the target compound was sequentially extracted with ethyl acetate; (2) partition coefficients (0.2 < *k* < 2) of the target compounds were suitable [17]. Based on these factors and with reference to previous research, a series of two-phase solvent systems containing *n*-hexane/*n*-butanol/water (1.5:5:5, 1.5:4:5, 1.5:3:5, 1.5:2:5 and 3:2:5 *v*/*v*/*v*) were tested, and the *k*-values of the target compounds were measured. However, none of the solvent systems could provide suitable *k*-values (Compounds **1**–**5**) between 0.2 and 2 at the same time, as shown in Table 1. Because of the wide polarity range of the target compounds, the crude extract could not be separated using a single solvent system. Therefore, two-step elution mode will be a good choice in the HSCCC separation. First, the two-phase solvent system *n*-hexane/*n*-butanol/water (1.5:5:5 *v*/*v*/*v*) was tested. The *k*-values of Compounds **4** and **5** were suitable, whereas they were unsuitable for target Compounds **1**–**3**. Therefore, the ratio of *n*-butanol should be decreased to increase the *k*-values of the target Compounds **1**–**3**. When the system was *n*-hexane/*n*-butanol/water (3:2:5 *v*/*v*/*v*), the *k*-values of Compounds **1**–**3** were suitable. Therefore, a two-step separation procedure was used for the separation of the five compounds. *n*-Hexane/*n*-butanol/water (1.5:5:5, *v*/*v*/*v*) was used for the first separation (Step 1) of Compounds **4** and **5**. *n*-Hexane/*n*-butanol/water (3:2:5, *v*/*v*/*v*) was used for the separation of Compounds **1**–**3** [18,19].

Moreover, this study demonstrated that the use of UPLC-PDA for the selection of a two-phase solvent system is an efficient method. It can quickly determine suitable partition coefficients (*k*).

Other factors, such as the flow rate of the mobile phase and the revolution speed of the separation column, were optimized. Different flow rates of the mobile phase (3.5, 5 and 10 mL/min) were tested to determine their effects on the separation time and peak resolution. The results indicate that a low flow rate resulted in a lengthy separation time with good peak resolution, whereas a high flow rate had the opposite effect. Therefore, a flow rate of 5 mL/min was selected to separate the target compounds. Moreover, the revolution speed affected the peak resolution. In this work, the revolution speed was set at 800 rpm, and good separation was observed.

### 2.3. HSCCC Separation of Sample

**Figure 1:**
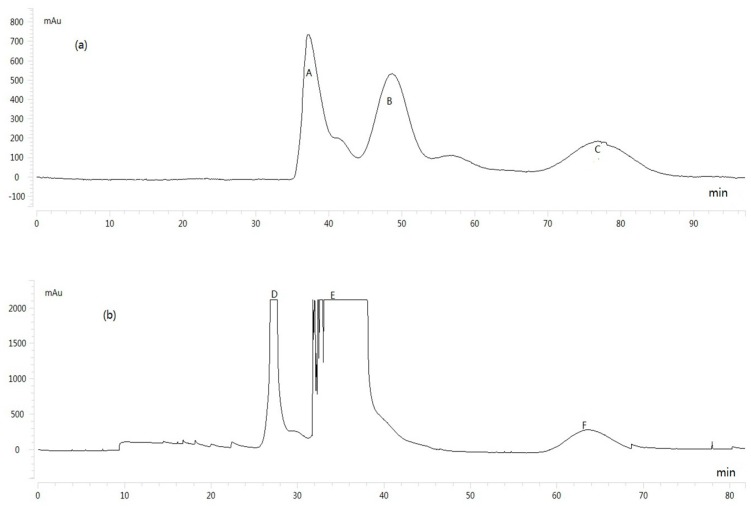
High-speed countercurrent chromatography (HSCCC) chromatogram of the ethyl acetate extract of *Veronica ciliata* Fisch. (**a**) First separation. Solvent system: *n*-hexane/*n*-butanol/water (1.5:5:5 *v*/*v*/*v*); mobile phase: upper phase; flow rate: 5 mL/min; revolution speed: rev 800 rpm; retention of stationary phase: 52%; sample size: 200 mg; (**b**) Second separation of Fraction A. Solvent system: *n*-hexane/*n*-butanol/water (3:2:5 *v*/*v*/*v*); mobile phase: upper phase; flow rate: 5 mL/min, revolution speed: rev 800 rpm; retention of stationary phase: 70%; sample size: 200 mg. (A–F represent HSCCC separation fractions).

**Figure 2:**
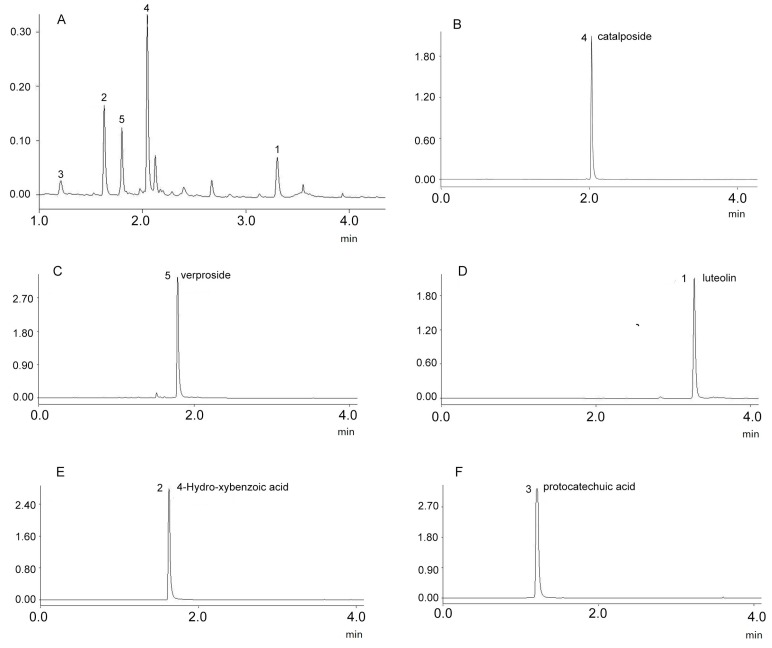
UPLC chromatograms of the sample (**A**) and the target compounds purified by HSCCC separation fraction (**B**–**F**).

The sample was separated into three fractions (A, B and C) using the *n*-hexane/*n*-butanol/water (1.5:5:5 *v*/*v*/*v*) solvent system. Then, the HSCCC peak fractions (D, E and F) were separated from the HSCCC Peak Fraction A using *n*-hexane/*n*-butanol/water (3:2:5 *v*/*v*/*v*), and the results are shown in Figure 1. The compounds with purity <90% were further purified by preparative HPLC. Notably, the purity of the five compounds analyzed by UPLC was over 98%, and the results are shown in Figure 2.

### 2.4. Structure Identification

The structures of the target compounds were determined by MS, ^1^H-NMR and ^13^C-NMR analyses. The results are as follows:

Compound **1**: Yellow powder, ESI-MS, *m*/*z* 287 [M + H]^+^. ^1^H-NMR (DMSO-*d*_6_, 600 MHz) δ 6.03 (1H, d, *J* = 2.1 Hz, H-6), 6.29 (1H, d, *J* = 2.1 Hz, H-8), 6.59 (1H, s, H-3), 6.76 (1H, d, *J* = 8.1 Hz, H-5′), 7.27 (1H, d, *J* = 2.1 Hz, H-2′), 7.29 (1H, dd, *J* = 8.1, 2.1 Hz, H-6′), 12.87 (1H, brs, 5-OH); ^13^C-NMR (100 MHz, DMSO-*d*_6_): see Table 2. Compared with the reported data [20,21], Compound **1** was identified as luteolin.

Compound **2**: White powder, ESI-MS, *m*/*z* 139 [M + H]^+^. δ: 7.793 (2H, d, *J* = 7.95 Hz, H-2,6), 6.81(2H, d, *J* = 7.95 Hz, H-3,5); ^13^C-NMR (100 MHz, DMSO-*d*_6_): see Table 2. Compared with the reported data [22], Compound **2** was identified as 4-hydroxy benzoic acid.

Compound **3**: White powder, ESI-MS, *m*/*z* 155 [M + H]^+^. ^1^H-NMR (600 MHz, DMSO-*d*_6_) δ 6.80 (1H, d, *J* = 8.5 Hz, H-5), 7.31 (1H, dd, *J* = 8.5 Hz, *J* = 1.8 Hz, H-6), 7.38 (1H, d, *J* = 2.0 Hz, H-2); ^13^C-NMR (100 MHz, DMSO-*d*_6_): see Table 2. Compared with the reported data [23], Compound **3** was identified as 3,4-dihydroxy benzoic acid.

Compound **4**: White powder, ESI-MS, *m*/*z* 505 [M + Na]^+^. ^1^H-NMR (600 MHz, DMSO-*d*_6_) δ 2.49 (1H, m, H-9), 2.57 (1H, m, H-5), 3.00–3.23 (4H, m, H-2′,3′,4′,5′), 3.42 (1H, dd, *J* = 11.8 Hz, 6.8 Hz, H-6′b), 3.68 (1H, d, *J* = 1.5 Hz, H-7), 3.71 (1H, dd, *J* = 11.8 Hz, 1.8 Hz, H-6′a), 3.72 (1H, d, *J* = 13.0 Hz, H-10b), 3.92 (1H, d, *J* = 13.5 Hz, H-10a), 4.63 (1H, d, *J* = 8.0 Hz, H-1′), 4.97 (1H, dd, *J* = 6.0 Hz, 4.5 Hz, H-4), 5.07 (1H, dd, *J* = 8.0 Hz, 1.0 Hz, H-6), 5.12 (1H, d, *J* = 9.5 Hz, H-1), 6.43 (1H, dd, *J* = 5.5 Hz, 1.5 Hz, H-3), 6.86 (2H, d, *J* = 9.0 Hz, H-3″,5″), 7.86 (2H, d, *J* = 8.5 Hz, H-2″,6′); ^13^C-NMR (100 MHz, DMSO-*d*_6_): see Table 2. Compared with the reported data [1], Compound **4** was identified as catalposide.

**Figure 3:**
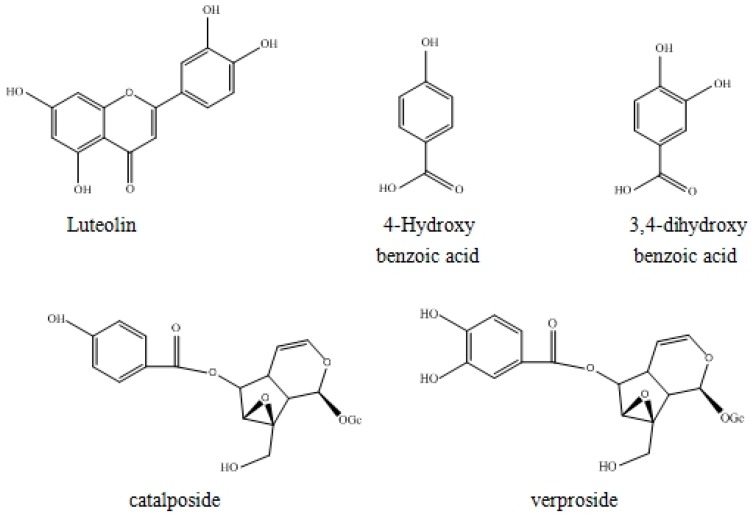
Structures of the five compounds.

Compound **5**: White powder, ESI-MS, *m*/*z* 521 [M + Na]^+^. ^1^H-NMR (600 MHz, DMSO-*d*_6_) δ 2.67 (1H, m, H-9), 2.69 (1H, m, H-5), 3.28–3.48 (4H, m, H-2″,3′,4′,5′), 3.69 (1H, dd, *J* = 12.2 Hz, 6.5 Hz, H-6′b), 3.78 (1H, d, *J* =1.0 Hz, H-7), 3.88 (1H, d, *J* = 13.5 Hz, H-10b), 3.97 (1H, dd, *J* = 12.0, 2.0 Hz, H-6′a), 4.21 (1H, d, *J* = 13.5 Hz, H-10a), 4.84 (1H, d, *J* = 7.5 Hz, H-1′), 5.03 (1H, dd, *J* = 6.0 Hz, 4.0 Hz, H-4), 5.13 (1H, dd, *J* = 8.0 Hz, 1.0 Hz, H-6), 5.23 (1H, d, *J* = 9.0 Hz, H-1), 6.41 (1H, dd, *J* = 6.3 Hz, 1.0 Hz, H-3), 6.86 (1H, d, *J* = 9.0 Hz, H-5″), 7.50 (1H, dd, *J* = 8.8 Hz, 2.3 Hz, H-6″), 7.51 (1H, d, *J* = 2.0 Hz, H-2″); ^13^C-NMR (100 MHz, DMSO-*d*_6_): see Table 2. Compared with the reported data [1], Compound **5** was identified as verproside. The structures of the five compounds are shown in Figure 3.

### 2.5. Antioxidant Activity

#### 2.5.1. DPPH• Scavenging Capacity

**Figure 4:**
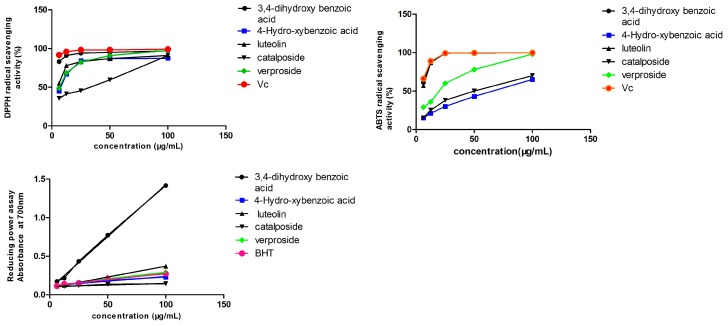
Determination of the antioxidant activity of the five compounds: (**A**) DPPH, (**B**) ABTS and (**C**) reducing power assay. Vc, vitamin C.

DPPH•, a stable radical, is often used to investigate the radical-scavenging capacity of natural products. Because of the existence of an unpaired electron, the DPPH radical shows a strong absorbance at 517 nm, providing a purple color. Upon the reaction with the antioxidant, the absorbance decreases due to the formation of the non-radical form, DPPH-H. Five samples of different concentrations (6.25, 12.5, 25, 50 and 100 μg/mL) and positive control vitamin C (Vc) were investigated for DPPH• scavenging capacity. The DPPH• scavenging ability of five compounds is shown in Figure 4. For each sample, the IC_50_ shows a concentration that causes a 50% reduction in DPPH radical concentration, as analyzed by GraphPad software. The IC_50_ values were small, indicating the strong antioxidant activity of the compounds. The IC_50_ values are shown in Table 3. The ability of the five compounds to scavenge the DPPH radical decreases in the order Vc > 3,4-dihydroxy benzoic acid > luteolin > verproside > 4-hydroxybenzoic acid > catalposide. All of the compounds have antioxidant activity, particularly protocatechuic acid, whose activity was equal to the activity of Vc.

#### 2.5.2. ABTS Radical-Scavenging Capacity

The ABTS cation radical is generated by the oxidation of ABTS with potassium persulfate. When the antioxidant is added, the radical is converted to the non-radical form. The ABTS radical-scavenging ability of five compounds is shown in Figure 4. Table 3 shows that the IC_50_ values of protocatechuic acid and luteolin were equal to that of Vc. This indicates that both protocatechuic acid and luteolin have strong antioxidant activity. The ABTS radical-scavenging ability was decreased in the order Vc > 3,4-dihydroxy benzoic acid > luteolin > verproside > catalposide > 4-hydroxybenzoic acid.

#### 2.5.3. Reducing Power Assay

The reducing power of the compounds was correlated with the potential antioxidant activity. To measure the reducing power of the five compounds, the ability to transform Fe^3+^ into Fe^2+^ was investigated. The reducing power of the five compounds is shown in Figure 4. The reducing power slope is shown in Table 3. The larger the slope, the better the reducing power. The order of the reducing power of the five compounds compared to BHT is as follows: 3,4-dihydroxy benzoic acid > luteolin > verproside > BHT > 4-hydroxybenzoic acid > catalposide. Protocatechuic acid, luteolin and verproside have a strong reducing power compared to the positive control BHT.

### 2.6. The Anti-Hepatocarcinoma Activity of Five Compounds

**Figure 5:**
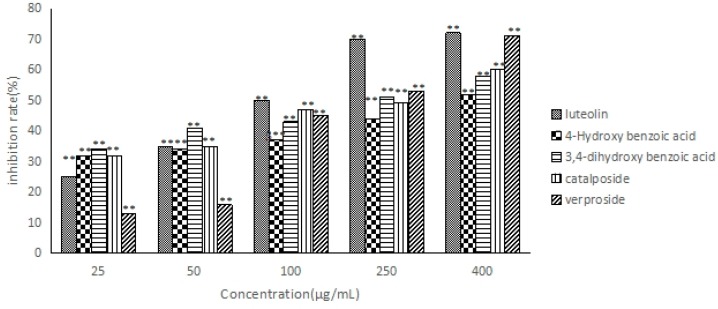
Cell proliferation inhibition rate of five compounds. ** *p* < 0.01, statistically significant in comparison with the control.

The five compounds were evaluated for their anti-hepatocarcinoma activity against HepG2 cell lines using the CCK-8 assay; these compounds all inhibited the proliferation of HepG2 cells (Figure 5); the inhibition rate increased in a concentration-dependent manner. According to the IC_50_ values (Table 4), the order of the anti-hepatocarcinoma activity of the five compounds is as follows: luteolin > verproside > catalposide > 3,4-dihydroxy benzoic acid > 4-hydroxybenzoic acid.

## 3. Discussion

According to the previous research, many antioxidants may have an important role in the hepatoprotective effect [2,24,25]. Therefore, in this experiment, we tested the relationship between the antioxidant and anti-hepatocarcinoma activity of the five compounds for the first time.

Firstly, the antioxidant activity of the five compounds was evaluated by DPPH, ABTS and the reducing power assay. The activities were in the order of 3,4-dihydroxy benzoic acid > luteolin > verproside > catalposide > 4-hydroxybenzoic acid.

The results demonstrated that the five compounds all have antioxidant activity when compared to Vc and BHT, especially luteolin and protocatechuic acid. This result was consistent with the previous research [26]. The antioxidant capacity of flavonoids (luteolin) was stronger than that of the iridoid glycosides (catalposide, verproside).

Secondly, the anti-hepatocarcinoma activities of five compounds were also evaluated against HepG2 cell lines by the CCK-8 method. The results show the proliferation of HepG2 cells was markedly inhibited by five compounds in a dose-dependent manner in vitro. The activities were in the order of luteolin > verproside > catalposide > 3,4-dihydroxy benzoic acid > 4-hydroxybenzoic acid. These strong and weak relationships were almost in line with the results of the antioxidant tests. Based on the previous publication [2,24,25] and our results, we can assume that the anti-hepatocarcinoma activity of the compounds, except 3,4-dihydroxy benzoic acid, is related, at least in part, to their antioxidant activity. In addition, we found that the anti-hepatocarcinoma activities were in the order of flavonoids (luteolin) > iridoid glycoside(catalposide, verproside) > phenolic acids (3,4-dihydroxy benzoic acid, 4-hydroxybenzoic acid). We suspected that this was because luteolin has more phenolic hydroxyl than other compounds [2,27]. Despite that the precise mechanisms of action of these phytochemicals are still unclear [28], this result has a significant effect on the further research.

## 4. Materials and Methods

### 4.1. HSCCC Apparatus

A TBE-300C HSCCC (Shanghai Tauto Biotech Co., Ltd., Shanghai, China) was coupled with a set of three multilayer coils and a 20-mL sample loop. The solvents were delivered using a TBP-5002 pump (Shanghai Tauto Biotech Co., Ltd.). A DC-0506 constant-temperature circulator (Shanghai Sunny Hengping Scientific Instrument Co., Ltd., Shanghai, China) was used to control the separation temperature. The UV absorbance of the eluent was monitored using a UV-2000 Detector (Shanghai Sanotac Scientific Instrument Co., Ltd., Shanghai, China) at a wavelength of 260 nm. The data were collected using an Easy Chrom v.2.2.1.23 workstation (Beijing Qingbohua Ltd., Beijing, China). The temperature of the separation column was maintained at 28 °C. A FB750D0-10A30 air compressor pump was used to remove the stationary phase.

### 4.2. Reagents and Materials

Acetonitrile (Swell Scientific Instruments Co. Ltd., Chengdu, China) was of chromatographic purity, and pure water (UPA, Chongqing, China) was used for UPLC. All of the reagents and solvents used for the preparation of the crude extract, HSCCC separation and antioxidant activities were of analytical grade (Kelun Chemical Reagent Co., Ltd, Chengdu, China). DMSO-d_6_ was used as the solvent for NMR analysis. The human hepatocellular (HepG2) cell line was purchased from ATCC (Rockefeller, MD, USA).

*Veronica ciliata* Fisch. herb was purchased from Tibet Tibetan Medicine Group Co., Ltd., China. A voucher specimen (No. 00721478) was identified by Jie Bai, School of Life Sciences, Sichuan University, and deposited in the Herbarium of Sichuan University.

### 4.3. Preparation of the Crude Extract

About 2 kg of dried *V. ciliata* Fisch. were powdered by a disintegrator. The powders were put in 25-L reagent barrels and were macerated with 95% ethanol (10 L) at room temperature. The extraction procedure was then repeated twice, each time for 24 h. The extracted solutions (26 L) were evaporated to dryness for 11 days, and the residues were dissolved in distilled water (0.5 L). Then, the solution was sequentially extracted ten times with petroleum ether (5 L) and three times with ethyl acetate (1.5 L). The combined ethyl acetate extracts were evaporated to dryness, affording 100 g of ethyl acetate extract for HSCCC separation.

### 4.4. Separation and Purification by HSCCC

#### 4.4.1. Selection of Two-Phase Solvent System

A two-phase solvent system was selected by considering the partition coefficient (*k*), which was determined using UPLC as follows:

The moderate crude sample was dissolved in an appropriate amount of a two-phase solvent system and placed in a separation funnel. The separation funnel was shaken vigorously to equilibrate the two-phase sample. Then, 1 mL of each phase was evaporated to dryness and dissolved in 2 mL methanol. The peak areas A1 and A2 were determined by UPLC, representing the target compounds in the upper and lower phases. The partition coefficient (*k*) was calculated using the formula *k* = A1/A2 [29].

#### 4.4.2. Preparation of Two-Phase Solvent System and Sample Solution

Mixtures of *n*-hexane/*n*-butanol/water (1.5:5:5) and *n*-hexane/*n*-butanol/water (3:2:5) were selected as the optimum two-phase solvent system; they were shaken and equilibrated in a separation funnel at room temperature. The upper and lower phases were separated and degassed by ultrasound for 30 min before use. A mixture of 200 mg ethyl acetate extract in 20 mL of the lower phase was prepared for the following experiment.

#### 4.4.3. HSCCC Separation

The column was first filled with the lower phase as the stationary phase at a flow rate of 30 mL/min. Subsequently, the upper phase was pumped into the inlet of the column as the liquid phase at a flow rate of 5 mL/min, while the apparatus was rotated at 800 rpm. The system reached equilibrium when the mobile phase eluted from the tail, and then, the retention of stationary phase was calculated. A solution of a 200-mg sample dissolved in 20 mL of the lower phase was injected into the column. The column temperature was maintained at 28 °C. Five main peaks were collected according to the chromatogram. The effluent was detected using a UV detector at 260 nm and evaporated to dryness. The residues were dissolved in methanol, and the sample solution was filtered through a 0.22-μm membrane filter before it was injected into the UPLC system. The fractions with purity <90% were further purified by preparative HPLC [30,31].

### 4.5. Purification of Compounds ***1**–**3*** by Preparative HPLC

The samples with purity <90% were further purified using a Waters 150 Preparative HPLC (Waters corporation, Milford, MA, USA) equipped with a binary solvent manager (Waters 2545), PDA detector and fraction collector. The prep-HPLC separation was performed as follows: a C18 column (250 mm × 10 mm, 5 μm) was used; the solvent system was 30% methanol; the eluent was pumped at 4 mL/min. The wavelength of the PDA detector was set to 260 nm.

### 4.6. Peak Identification

The peaks obtained from the HSCCC of the target compounds were determined by UPLC-PDA, MS, ^1^H-NMR and ^13^C-NMR spectra.

#### 4.6.1. UPLC-PDA Analysis

UPLC analysis was performed using a Waters ACQUITY UPLCTM system (Waters Corporation) equipped with a quaternary solvent manager system, autosampler and a PDA detector. The samples were eluted using a Waters ACQUITYUPLC HSS T3 column (100 × 2.1 mm, 1.7 μm). The column and autosampler temperatures were maintained at 40 °C and 25 °C, respectively. A mobile phase containing water (A) and ACN (B) was used with the optimized gradient program as follows: 10%–25% B (0–0.8 min), 25%–35% B (0.8–2.4 min), 35%–95% B (2.4–2.8 min), 95% B (2.8–4 min). The flow rate was set at 0.5 mL/min. The sample injection volume was 1 μL. The wavelength of PDA detector was set to 260 nm. Empower software Version 3.0 (Waters Corporation) was used for system control and data acquisition [32].

#### 4.6.2. MS Analysis

ESI-MS spectra (Waters Corporation) were recorded using a micrOTOF-Q II 10203 triple quadrupole mass spectrometer equipped with electrospray ionization and operated in the positive ionization mode. The Bruker Compass Data Analysis 4.0 software (Waters Corporation) was used to analyze the data.

#### 4.6.3. NMR Analysis

The ^1^H-NMR spectra and ^13^C-NMR spectra were recorded using a Bruker Ascend-600 spectrometer (Bruker Co., Karlsruhe, Germany) operated at 600 MHz and 100 MHz for ^1^H- and ^13^C-NMR, respectively, using DMSO-*d*_6_ as the solvent. The chemical shifts were reported in δ (ppm) downfield from tetramethylsilane (TMS) as the internal standard, and the coupling constants were reported in Hz.

### 4.7. In Vitro Antioxidant and Anti-Hepatocarcinoma Activity

#### 4.7.1. DPPH• Scavenging Capacity [33]

The DPPH• scavenging free radical test was performed following the previous study with minor modification. A solution of DPPH in ethyl alcohol (100 μL, 0.1 mmol) was added to different concentrations of 100-µL samples of Vc (100, 50, 25, 12.5 and 6.25 µg/mL). The mixture was shaken and incubated for 30 min at room temperature. The absorbance values were measured at 517 nm using a spectrophotometer (Thermo Fisher Scientific Oy, Ratastie 2, Vantaa, Finland). Ethyl alcohol was used as the control. Vc was used as the positive control. The results were calculated using the following formula:

(1) DPPH• scavenging activity (%) = [1−(Ai−As)/Ac] × 100

where Ai, As and Ac represent the absorbance of the sample with DPPH, the background of the sample and the control.

#### 4.7.2. ABTS Radical-Scavenging Capacity

The ABTS radical-scavenging capacity of the five compounds was determined following the methods used in the previous study [34]. The ABTS^+^ solution was prepared by reacting 7 mM ABTS and 2.45 mM potassium persulfate at room temperature in the dark for 12 h. The solution was then diluted with phosphate buffer (pH 7.6) to acquire an absorbance of 0.7 ± 0.02 at 734 nm. Further, 100-μL samples (6.25–100 μg/mL) were mixed with 100 µL ABTS^+^ solution. The mixtures were incubated at 25 °C for 30 min, and the absorbance was measured at 734 nm using a microplate reader. The formula of DPPH was fitted for the calculation of the ABTS radical-scavenging capacity. Vc was used as the positive control.

#### 4.7.3. Reducing Power Assay [35]

The reducing power assay of the samples was measured following the previous method with minor modifications.

First, 25 μL of different concentrations (6.25, 12.5, 25, 50 and 100) of the sample or BHT dissolved in methanol were added to 50 μL PBS (pH 6.6, 0.2 M) and 25 μL of a 1% (*w*/*v*) K_3_Fe(CN)_6_ solution. The mixture was shaken and incubated for 30 min at 50 °C. Next, 50 μL of 10% (*w*/*v*) trichloroacetic acid (TCA) were added, followed by 60 μL of 1% (*w*/*v*) FeCl_3_. The absorbance of the mixture was measured at 700 nm.

BHT was used as the positive control. A high absorption value indicated a higher reducing power.

#### 4.7.4. In Vitro Anti-Hepatocarcinoma Activity [2,12]

##### Cell Culture

Human hepatocellular carcinoma HepG2 cells were obtained from ACTT.

The cells were cultured in DMEM medium (Gibco BRL) containing 10% fetal bovine serum (FBS), 100 IU/mL penicillin and 100 IU/mL streptomycin under humidified air with 5% CO_2_ at 37 °C.

##### Cell Proliferation Inhibition Assay

The effect of each sample on the proliferation of HepG2 cells was estimated using the CCK-8 test. HepG2 cells in the exponential growth phase, at a density of 5 × 10^4^ cells/mL, were added in 96-well culture plates (100 µL/well) and incubated overnight. The cells were treated with different concentrations of samples. After 48 h, the medium was renewed, treated with 10 µL CCK-8 solution and incubated for an additional 2 h. The optical density (OD) of the solution was measured at 450 nm using a microplate reader. The positive control was 5-fluorouracil. The cell proliferation inhibition rate (CPIR) was identified and calculated using the following formula:

(2) Cell proliferation inhibition rate = [1 − ODsample/ODcontrol] × 100

### 4.8. Statistical Analysis

All of the experiments were conducted in triplicate and the results expressed as the mean ± standard deviation (SD). Statistical differences of experimental data among groups were tested using one-way ANOVA (*n* = 3) analysis or paired two-sample *t*-test (*n* = 3) analysis (SPSS 15.0, SPSS Inc., Chicago, IL, USA). *p* < 0.05 was considered to be statistically significant.

## 5. Conclusions

In this report, an effective method was successfully established to separate five compounds with purity over 98% from the crude extract of *Veronica ciliata* Fisch. The results of our study could demonstrate the following two points: (1) the two-step elution for the separation of different polarities of target compounds by HSCCC is an efficient strategy [19]; (2) the above results demonstrate that these compounds were the active chemical components responsible for the antioxidant and anti-hepatocarcinoma properties of *V. ciliata* Fisch., and the anti-hepatocarcinoma activity of the five compounds is related, at least in part, to their antioxidant activity. The underlying mechanism of their anti-hepatocarcinoma activity is worthy of further investigation [2,19].

In future, these phenolic compounds and iridoid glycosides or their combinations may be developed as hepatoprotective drugs for use in human liver disease. Additionally, *V. ciliata* Fisch., as a traditional Tibetan medicine, which possesses strong antioxidant and anti-hepatocarcinoma activity, may play a significant role in the treatment of liver disease.

## Figures and Tables

**Table 1:** *k*-values of five compounds in various solvent system.

Solvent System (*n*-Hexane/*n*-Butanol/Water)	*k*-Values				
	*k*1	*k*2	*k*3	*k*4	*k*5
1.5:5:5	0.009	0.13	0.19	0.52	1
1.5:4:5	0.019	0.17	0.55	0.56	1.7
1.5:3:5	0.02	0.19	0.59	0.67	2.1
1.5:2:5	0.1	0.2	0.6	1.4	3.8
3:2:5	0.21	0.32	0.95	2.7	5.8

**Table 2:** ^13^C-NMR (100 MHz, DMSO-*d*_6_) of the five compounds.

Carbon	Compound				
	1	2	3	4	5
1		121.95	123.8	92.9	95.6
2	164.2	131.98	116.6		
3	103.6	115.56	146.8	141.2	142.8
4	182.4	162.02	152.0	101.6	103.6
5	162.7	115.56	118.6	35.3	36.8
6	99	131.98	124.8	80.1	82.1
7	164.5		170.9	58.3	59.9
8	94			65.7	67.2
9	158.1			41.7	43.7
10	104.7			58.5	61.8
1′	123.1			97.8	99.6
2′	113.5			73.3	75.6
3′	145.8			77.5	78.5
4′	149.4			70.4	72.4
5′	116			76.5	77.5
6′	119.5			61.5	63.9
1″				119.4	122.4
2″				131.6	116.6
3″				115.4	146.4
4″				162.4	152.4
5″				115.4	117.4
6″				131.6	124.6
7″				165.5	168.8

**Table 3:** The antioxidant activity of the five compounds.

Samples	IC_50_ (μg/mL)		Reducing Power
	DPPH	ABTS	
luteolin	4.168	5.587	0.0028
4-hydroxy benzoic acid	6.838	58.78	0.0012
3,4-dihydroxy benzoic acid	0.703	5.186	0.013
catalposide	20.16	43.83	0.0004
verproside	6.502	16.9	0.0018
Vc	0.57	4.645	
BHT			0.0016

**Table 4:** Anti-hepatocarcinoma activity of target compounds (IC_50_, μg/mL).

Compounds	HepG2
luteolin	102.356 ± 2.01
4-hydroxy benzoic acid	444.759 ± 2.65
3,4-dihydroxy benzoic acid	186.033 ± 2.27
catalposide	184.592 ± 2.27
verproside	177.147 ± 2.25
5-FU	35.420 ± 1.56
